# Satellitome Analysis in the Southern Lapwing (*Vanellus chilensis*) Genome: Implications for SatDNA Evolution in Charadriiform Birds

**DOI:** 10.3390/genes15020258

**Published:** 2024-02-19

**Authors:** Rafael Kretschmer, Gustavo A. Toma, Geize Aparecida Deon, Natalia dos Santos, Rodrigo Zeni dos Santos, Ricardo Utsunomia, Fabio Porto-Foresti, Ricardo José Gunski, Analía Del Valle Garnero, Thomas Liehr, Edivaldo Herculano Corra de Oliveira, Thales Renato Ochotorena de Freitas, Marcelo de Bello Cioffi

**Affiliations:** 1 Departamento de Ecologia, Zoologia e Genética, Universidade Federal de Pelotas, Pelotas 96010-900, RS, Brazil; rafael.kretschmer@ufpel.edu.br; 2 Departamento de Genética e Evolução, Universidade Federal de São Carlos, São Carlos 13565-905, SP, Brazil; gustavo_toma@hotmail.com (G.A.T.); geizedeon@hotmail.com (G.A.D.); mbcioffi@ufscar.br (M.d.B.C.); 3 Faculdade de Ciências, Universidade Estadual Paulista, Bauru 13506-900, SP, Brazil; n.santos97@unesp.br (N.d.S.); rodrigo.zeni@unesp.br (R.Z.d.S.); ricardo.utsunomia@unesp.br (R.U.); fp.foresti@unesp.br (F.P.-F.); 4 Laboratório de Diversidade Genética Animal, Universidade Federal do Pampa, São Gabriel 97300-162, RS, Brazil; ricardogunski@unipampa.edu.br (R.J.G.); analiagarnero@unipampa.edu.br (A.D.V.G.); 5 Institute of Human Genetics, Friedrich Schiller University, University Hospital Jena, 07747 Jena, Germany; 6 Laboratório de Citogenô mica e Mutagênese Ambiental, Seção de Meio Ambiente, Instituto Evandro Chagas, Ananindeua 67030-000, PA, Brazil; ehco@ufpa.br; 7 Instituto de Ciências Exatas e Naturais, Universidade Federal do Pará, Belém 66075-110, PA, Brazil; 8 Laboratório de Citogenética e Evolução, Departamento de Genética, Instituto de Biociências, Universidade Federal do Rio Grande do Sul, Porto Alegre 91509-900, RS, Brazil; thales.freitas@ufrgs.br

**Keywords:** Charadriidae, chromosome evolution, constitutive heterochromatin, sex chromosomes, repetitive DNA

## Abstract

*Vanellus* (Charadriidae; Charadriiformes) comprises around 20 species commonly referred to as lapwings. In this study, by integrating cytogenetic and genomic approaches, we assessed the satellite DNA (satDNA) composition of one typical species, *Vanellus chilensis*, with a highly conserved karyotype. We additionally underlined its role in the evolution, structure, and differentiation process of the present ZW sex chromosome system. Seven distinct satellite DNA families were identified within its genome, accumulating on the centromeres, microchromosomes, and the W chromosome. However, these identified satellite DNA families were not found in two other Charadriiformes members, namely *Jacana jacana* and *Calidris canutus*. The hybridization of microsatellite sequences revealed the presence of a few repetitive sequences in *V. chilensis*, with only two out of sixteen displaying positive hybridization signals. Overall, our results contribute to understanding the genomic organization and satDNA evolution in Charadriiform birds.

## 1. Introduction

The extensive chromosomal variation found in birds makes them an excellent group for investigating evolutionary mechanisms responsible for karyotype reorganization and stability [1,2,3,4]. For these kinds of investigations, birds from Charadriiformes constitute an outstanding model. By comparing with the proposed ancestral avian karyotype, which is thought to have had 80 chromosomes [1], this group presents species with both conserved and highly rearranged karyotypes. Most studies on karyotype evolution and the effects of chromosomal rearrangements in Charadriiformes species have used conventional cytogenetics and chromosome painting techniques [5,6,7,8,9]. For instance, the southern lapwing (*V. chilensis*) exhibits a karyotype with a diploid number (2n) of 2*n* = 78, with only one interchromosomal rearrangement resulting from the fusion of chicken (*Gallus gallus*) chromosomes 7 and 8 [7]. On the other hand, the wattled jacana (*J. jacana*) has a 2*n* = 82 karyotype, but six fusion events and eight fissions involving chicken chromosomes 2, 3, 4, 5, 6, 7, 8, and a pair of microchromosomes have been described [8]. Further research has demonstrated that microchromosomes 11–28 are conserved in both species, according to thorough examinations utilizing bacterial artificial chromosome (BAC) probes [10].

More recently, the integration of cytogenetics with high-throughput sequencing data from next-generation sequencing (NGS) technologies has allowed a new perspective, allowing the characterization of satDNA families within species [11,12,13]. This comprehensive compilation, often referred to as the “satellitome”, has yielded invaluable insights into various evolutionary aspects. These insights encompass karyotype evolution, genome diversity, phylogenetic relationships, as well as the evolution and structure of sex chromosomes, and their contributions to speciation processes [14,15,16,17]. However, the arrangement of satDNA sequences on chromosomes in birds is yet mainly unknown since most studies on the subject concentrated on specific species or satDNA families [18,19,20,21,22,23,24]. Recently, a few works focusing on the avian satellitome have been conducted [25,26,27,28].

The genus *Vanellus* (Charadriidae, Charadriiformes) contains 23 valid species commonly known as lapwings, primarily found along lakes and riverbanks or in open grasslands [29]. Charadriidae is known for its low karyotype variability, with 2*n* ranging from 72 to 78 [7,9,30,31,32,33,34,35]. Their sex chromosomes follow the usual avian pattern, with the Z chromosome being larger than the W, which is normally small and heterochromatic [7,30,31,32,33,34,35].

In contrast, there is significant chromosomal variability in other families in the order Charadriiformes. While *C. canutus* (Scolopacidae) presents a 2*n* = 92 [10], one member of the Burhinidae family, i.e., *Burhinus oedicnemus*, has a karyotype with an uncommonly low diploid number, 2*n* = 42, due to extensive chromosomal fusions, especially involving the microchromosomes [5]. Nonetheless, *J. jacana*, a member of the Jacanidae family, presents 2*n* = 82 but with several chromosomal rearrangements, particularly involving the fusion and fission of macrochromosomes [8].

In this study, we coupled cytogenetic and genomic approaches to analyze the satellitome composition of *V. chilensis*. We aim to investigate the potential role of these satDNAs and microsatellite repeats in the sex chromosome evolution and the large chromosome reshuffling experienced by this species. Additionally, we compared the intragenomic differences between males and females through comparative genomic hybridizations and also mapped the satDNA families found in *V. chilensis* in two other species of Charadriiformes named *J. jacana* (Jacanidae) and *C. canutus* (Scolopacidae) to obtain insights into the evolutionary history of satDNA families in birds, particularly in the order Charadriiformes.

## 2. Materials and Methods

### 2.1. Samples, Chromosome Preparations, and C-Banding

Our sampling, composed of two individuals of *V. chilensis*, three *J. jacana*, and two *C. canutus* (Table 1), was conducted under the Brazilian Environmental Agency ICMBIO/SISBIO (61047-4 and 44173-1) and the Ethics Committee on Animal Use (CEUA) of the Federal University of Pampa (018/2014 and 019/2020). The chromosomal preparations were obtained from fibroblast cell cultures using skin biopsies from each individual following the protocol described by Sasaki et al. [36]. The cells were cultured in culture flasks (25 cm^2^) with DMEM culture medium (GIBCO), supplemented with fetal bovine serum (15% GIBCO) and 1% Penicillin (10,000 units/mL)/streptomycin (10,000 μg/mL) (GIBCO), and incubated at 37 °C. Metaphase chromosomes were obtained through treatment with colchicine (1 h, 37 °C), hypotonic solution (0.075 M KCl, 15 min, 37 °C), and fixation with methanol/acetic acid (3:1). The same chromosome preparations analyzed in this study have been used recently by Oliveira et al. [26]. C-banding was performed according to Sumner [37] with some modifications according to Lui et al. [38].

### 2.2. DNA Extraction and Genome Sequencing

The genomic DNA (gDNA) of a male and female individual of *V. chilensis* was extracted using the phenol/chloroform-based protocol reported in Sambrook and Russel [39]. The samples were sequenced using the BGISEQ-500 platform (paired-end 2 × 150 bp) and yielded a total size of 2.44 Gb and 2.43 Gb for females and males, respectively. The obtained reads were deposited on the Sequence Read Archive (SRA-NCBI) and are available under the accession numbers SRR27919084 (male) and SRR27919083 (female).

### 2.3. Characterization of Satellitome and BLAST Search of VchSatDNAs

The satellitome of males and females of *V. chilensis* were independently characterized by the Tandem Repeat Analyzer (TAREAN) tool [40]. We performed an initial assessment of the read quality (Q > 20 for all nucleotides) by using the trimmomatic software version 3 [41]. Then, as an input for the TAREAN clustering [40], we selected a random subset of 2 × 500,000 reads. The satDNAs recovered were filtered by the DeconSeq software version 0.4.3 [42] from the original subset of 2 × 500,000 reads, which were further loaded in TAREAN until no new satDNAs were identified. We removed other types of repetitive DNAs (e.g., multigene families) from the *V. chilensis* satellitome and assembled them into variants (>95% similar), families (80–95% similar), and superfamilies (50–80% similar) by using RM_Homology (https://github.com/fjruizruano/satminer/blob/master/rm_homology.py (accessed on 19 December 2023)) and Geneious software version 8.1 [11]. Finally, to estimate the abundance (mapped reads divided by the number of total nucleotides) and divergence (Kimura-2-parameter) of VchSatDNAs, we used a combination of a custom python script (https://github.com/fjruizruano/ngs-protocols/blob/master/repeat_masker_run_big.py, accessed on 20 December 2023) and the RepeatMasker softwareversion 4.1.6 [43]. Specifically, we selected 2 × 5,000,000 reads of each genomic library (male and female) and aligned them against their satDNA catalogs. Each VchSatDNA was named and numbered following the suggestions from Ruiz-Ruano et al. [11] and further represented in the form of repeat landscapes. To check for putative female-biased satDNAs, we calculated the female/male (F/M) abundance ratio of each VchSatDNA. Finally, we conducted a BLAST search [44] of all VchSatDNAs against the GenBank/NCBI (nucleotide collection) and Repbase databases (CENSOR) to verify the sharing of conserved DNA motifs with other closely related species and repetitive DNA sequences.

### 2.4. Primer Design and VchSatDNAs Amplification by Polymerase Chain Reaction (PCR)

We designed primers for six out of seven VchSatDNAs. The primers were manually designed for each consensus monomer, using multiple primer analyzer (Thermofisher) and OligoCalc (Biotools) to check for hairpin formation and self-annealing. Due to its small size (<30 pb), VchSat07-36 was directly synthesized and labeled with Biotin at the 5’ end by ThermoFisher (ThermoFisher Scientific, Waltham, MA, USA). The VchSatDNAs were amplified by PCR, using, in general, a starting denaturation step of 95 °C for 5 min, 30 cycles with 95 °C for 20 s, 39.1–54.2 °C as the annealing temperature for 40 s, 72 °C for 30 s, and a final extension step of 10 min following Kretschmer et al. [16]. The PCR products were quantified using a ThermoFisher NanoDrop spectrophotometer (ThermoFisher Scientific) and submitted to electrophoresis with a 1% or 2% agarose gel.

### 2.5. Fluorescence In Situ Hybridizations (FISH)

All VchSatDNAs were labeled using a nick translation kit from Jena Bioscience (Jena, Germany) incorporating the fluorophore Atto550-dUTP according to the instructions in the manufacturer’s manual. The microsatellite sequences (GAA)_n_, (GAC)_n_, (CGG)_n_, (CAC)_n_, (CAG)_n_, (CAT)_n_, (GAG)_n_, (TAA)_n_, (TAC)_n_, (CAA)_n_, (GA)_n_, (CA)_n_, (GC)_n_, (TA)_n_, (C)_n_, and (A)_n_ were labeled directly with Cy3 at the 5’ end during synthesis (VBC Biotech, Vienna, Austria) and also used in the fluorescence in situ hybridization (FISH) experiments. We performed the FISH experiments following the protocol described by Kretschmer et al. [45]. Briefly, after one hour of aging at 60 °C, chromosomal preparations were treated for one hour and thirty minutes with an RNAse solution (1.5 µL RNase A (10 mg/mL) in 1.5 mL 2× SSC). After that, chromosomes were treated with a pepsin solution containing 0.005% (*v*/*v*) (2.5 µL pepsin (20 mg/mL), 10 µL 1M HCl, and 99 µL H_2_O). For two minutes, slides were denatured in 70% formamide in 2× SSC at 72 °C. The probe mix per each slide contained 200 ng of probe dissolved in the hybridization buffer comprising 10% dextran sulfate, 50% formamide, and 2× SSC. This mix was subsequently applied onto denatured chromosome slides after being denatured for ten minutes at 86 °C. After 72 h of hybridization in a dark and humid chamber at 37 °C, a series of post-hybridization washes in 1× SSC, 4× SSC/Tween, and 1× PBS solutions were conducted. The slides were finally dehydrated in 70%, 85%, and 100% ethanol solutions, and the metaphases were stained with 4′,6-diamidino-2-phenylindole (DAPI).

### 2.6. Comparative Genomic Hybridization (CGH): Experimental Design and Probe Preparation

The genomic DNAs of *V. chilensis* male and female specimens were isolated from feather pulp tissues using the conventional phenol–chloroform procedure [39]. Using nick-translation (Jena Biosciences), the gDNAs of the male and female specimens were labeled using Atto550-dUTP and Atto488-dUTP, respectively. These probes were then hybridized to the female chromosomal complement. We employed unlabeled C0t-1 DNA, a gDNA fraction enriched in highly and moderately repetitive sequences, synthesized following [46], to block the common repetitive sequences. The final hybridization mixtures contained 500 ng of male gDNA (labeled with Atto550-dUTP) plus 500 ng of female gDNA (labeled with Atto488-dUTP) supplemented with 3 μg of male-derived C0t-1 DNA dissolved in 20 μL of the hybridization buffer (50% formamide, 2× SSC, 10% SDS, 10% dextran sulfate, and Denhardt’s buffer, pH 7.0). The ratio of the probe vs. C0t-1 DNA was chosen based on previous investigations [47,48,49]. The slides were aged at 37 °C for two hours before hybridization occurred. They were then treated with RNAse A for 90 min at 37 °C and pepsin for three minutes at 37 °C (50 μg/mL in 10 mM HCl). Then, the chromosomes were quickly cooled and dehydrated using a sequence of 70% (cold), 85%, and 100% (RT) ethanol after being denatured in 75% formamide (pH 7.0) in 2× SSC (74 °C, 3 min). After being denatured for six minutes at 86 °C, the hybridization mixture was applied to the slides and cooled for ten minutes at 4 °C. The hybridization was placed for 72 h at 37 °C. Three post-hybridization washes in 1× SSC (44 °C, 7 min each) and one in 50% formamide in 2× SSC (pH 7.0) were performed. Chromosomes were counterstained and mounted in antifade containing 1.5 μg/mL DAPI (Vector, Burlingame, CA, USA).

## 3. Results

### 3.1. Cytogenetic Data

Our data corroborated previous karyological information for these species described by Kretschmer et al. [7], Kretschmer et al. [8], and de Souza et al. [10]. In summary, despite belonging to the same order, these species display diverse 2*n*: *V. chilensis* (2*n* = 78), *J. jacana* (2*n* = 82), and *C*. *canutus* (2*n* = 92). We described here, for the first time, the W sex chromosome of *V. chilensis*, which is an acrocentric medium-sized chromosome. C-banding shows that the C-positive heterochromatin on this species is located mainly on the short arms of the W chromosome and in some microchromosome pairs.

### 3.2. General Characterization of VCH Satellitome

After three iterations of TAREAN repeat clustering, we recovered seven VchSatDNA families (Table 2), available under the accession numbers PP135771 to PP135777.

**Figure 1:**
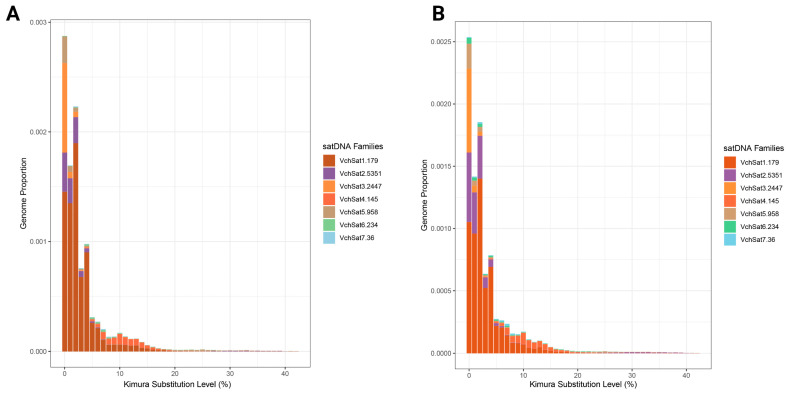
Repeat landscape of male (**A**) and female (**B**) *V. chilensis* showing abundance (Y axis) and Kimura-2-divergence (X axis) of all uncovered VchSatDNAs.

The repeat landscapes of both male and female are presented in Figure 1. Most VchSatDNAs presented an A + T (%) lower than 50%, except for only three satDNAs. The repeat unit lengths (RUL) ranged from 36 bp to 5351 bp (average of 1336 bp). Additionally, 86% of VchSatDNAs displayed RULs greater than 100 bp, with VchSat07-36 being the only shorter satDNA (<100 bp) (Table 2). By calculating the sex abundance ratio (F/M), we found three female-biased VchSatDNAs (VchSat 02, VchSat06-234, and VchSat07-36) (Table 2). The BLAST search against GenBank/NCBI revealed a strong similarity (Query cover = 77%; Percentage identity = 88%; E-value = 4 × 10^−35^) between VchSat01-179 and a centromeric tandem repeat (187 pb) found in *Vanellus spinosus* (GenBank access number: S72324.1). By searching against “all sources” in Repbase, we found five VchSatDNA (VchSat01-03; VchSat05; VchSat06) embedded with other repetitive DNA sequences, namely transposons, endogenous retrovirus sequences (ERVs), and retrotransposons (Figure S1). For the remaining VchSatDNAs, however, no significant contig matches were observed. We found no evidence of any superfamily (SF) relationship among VchSatDNAs and between *V. chilensis* and *J. jacana* satDNAs.

### 3.3. Chromosomal Distribution of VchSatDNAs and Microsatellites

**Figure 2:**
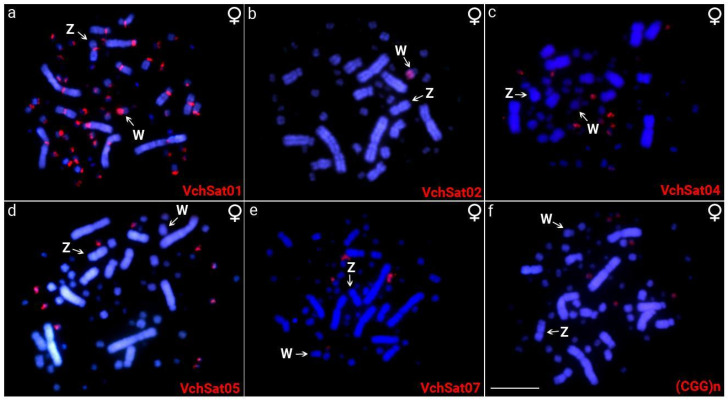
Mitotic chromosome spreads of *V. chilensis* (VCH) female individuals after FISH using VchSatDNAs (**a**–**e**) and (CGG)_n_ microsatellite (**f**) probes. Their family names are indicated in the lower right corner in red (Atto550-dUTP labeled). The Z chromosome was assigned arbitrarily based on its morphology, representing an unpaired submetacentric macrochromosome, whereas the W chromosome was properly recognized by a sequential hybridization with the microsatellite (GAA)n. The Z and W sex chromosomes are also indicated in each metaphase. Scale bar = 20 μm.

**Figure 3:**
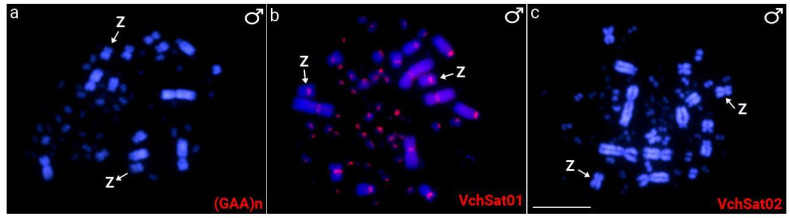
Mitotic chromosome spreads of *V. chilensis* (VCH) male individuals after FISH using (GAA)_n_ microsatellite (**a**) and VchSatDNAs (**b**,**c**) probes. Their family names are indicated in the lower right corner in red (Atto550-dUTP labeled). The Z chromosomes were assigned arbitrarily based on its morphology, representing a large submetacentric macrochromosomal pair. Scale Bar = 20 μm.

Except for VchSat03-2447 and VchSat06-234, all the other VchSatDNAs obtained through PCR effectively hybridized to *V. chilensis* female chromosomes by FISH, demonstrating that they are mostly arranged in long and high copy number arrays in the genome (Figure 2 and Figure 3). VchSat01-179 was conspicuously located at the centromeres of all chromosomes, including autosomes and sex chromosomes (Figure 1A). VchSat02-5351 was explicitly located on the long arms of the W chromosome (Figure 1B). VchSat04-145, VchSat05-958, and VchSat07-36 produced FISH signals exclusively in multiple microchromosomes (Figure 2). Although VchSat07-36 exhibits the highest F/M abundance ratio (Table 2), it produces FISH signals, but none of them are on the W chromosome (Figure 2e), probably because it is low in abundance and only makes up a small fraction of the genome.

**Figure 4:**
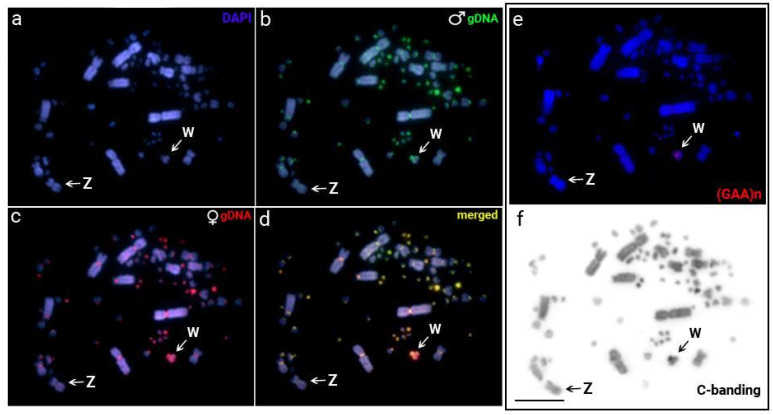
Intraspecific genomic hybridization using male (green) and female (red) genomic DNA probes from *V. chilensis* (VCH) hybridized on female metaphasic chromosomes. (**a**) DAPI staining (blue), (**b**) hybridization pattern of the male-derived probe (green), (**c**) hybridization pattern of the female-derived probe (red), and (**d**) merged images of both genomic probes and DAPI staining. The common regions for male and female genomes are depicted in yellow. Sequential hybridization with (GAA)n probe (**e**) and C-banding (**f**) performed to correctly identify the W chromosome are boxed. The Z chromosome was assigned arbitrarily based on its morphology, representing an unpaired submetacentric macrochromosome. The Z and W sex chromosomes in metaphase are indicated in each slide. Scale bar = 20 μm.

Furthermore, we investigated by FISH the presence of VchSatDNAs in two additional members of the Charadriiformes order, namely *J. jacana* and *C*. *canutus*. Nevertheless, no hybridization signal could be observed. Of the total of microsatellites tested, only two had positive hybridization signals in *V. chilensis*, i.e., (GAA)_n_ and (CGG)_n_. While the first one was exclusively accumulated on the W chromosome, the second displayed clusters in three pairs of microchromosomes (Figure 2, Figure 3 and Figure 4).

### 3.4. Intraspecific Comparative Genomic Hybridizations (CGH)

Almost all chromosomes had overlapping signals in their pericentromeric regions, except for a significant female-specific region on the W chromosome (Figure 4d) that coincided with a C-positive heterochromatic block (Figure 4f).

## 4. Discussion

Our findings align with the hypothesis that, compared to other vertebrates, birds have more compacted genomes with fewer repetitive DNAs [50]. Using bioinformatic techniques, we characterize the satellitome of *V. chilensis* and, as a result, seven VchSatDNA families are present in this species (Table 2). A similarly low number of satDNAs (11) were found for the *J. jacana* genome, another Charadriiform species [24]. In fact, the overall set of satDNAs found in birds is relatively small compared to investigations carried out across other animal taxa [13,16,51].

Notably, the size of repeat units (RUL) in the *V. chilensis* genome exhibited a range from 36 to 5351 base pairs, with longer satellites (>100 bp) predominating over those considered shorter (<100 bp) (Table 2). Satellites with larger repeat units emerge as a distinct characteristic within bird genomes, setting them apart from species in other groups that tend to feature shorter satellites [13,25,52,53,54]. The number of satellite DNA families present in genomes is very diverse. The species with the most identified sequences is the crayfish *Pontastacus leptodactylus* (Astacidae), which has 258 satellites [54]. Furthermore, four out of the seven VchSatDNA sequences had a much greater GC content (>50%), a tendency that may be common among bird species [25,26].

Our research identified VchSat01-179 as a potential candidate for the centromeric satDNA. It is present within the centromeres across all chromosomes, including autosomes and sex chromosomes. VchSat01-179 comprises GC-rich regions, which make up 52.6% of this satDNA’s sequences. This is in contrast to the fact that centromeric satDNA is often AT-rich, as seen in many different species [55,56,57]. It has been suggested that centromeric repeats represent one of the fastest-evolving DNA sequences within eukaryotic genomes [58,59,60]. This rapid evolution mechanism within centromeric sequences has been proposed to be associated with the speciation process. Such evolutionary changes can potentially contribute to reproductive isolation and, ultimately, the diversification of species [58,61,62]. Our BLAST analysis against GenBank/NCBI databases revealed a tandem repeat of 187bp, resembling VchSat01-179, in the genome of *V. spinosus* [63]. The authors suggest that conserved oligonucleotide motifs of this tandem repeat are shared between other bird species (e.g., *Phoenicopterus chilensis*, *Grus antigone*, *Falco columbarius*) and likely originated from a common ancestor [63]. However, our FISH experiments indicated that VchSat01-179 may be specific to *V. chilensis* as it was not detected in both *J. jacana* and *C*. *canutus* species. In addition, the satellite analysis conducted on *J. jacana* did not identify the presence of a specific satDNA across all chromosomes [26]. Another possibility is that this satDNA sequence is present in more closely related species, such as other lapwing species. Further research is necessary to determine whether other congeneric species also share their motifs and whether it is their major centromere component.

Our study revealed that most VchSatDNAs were predominantly localized within the microchromosomes, specifically with VchSat04-145, VchSat05-958, and VchSat07-36 likely residing within heterochromatic microchromosomes (Figure 2). Avian microchromosomes are generally known for their GC-rich nature [64]. Supporting this notion, except for the VchSat05-958, which has an amount of 52.4% of AT, VchSat04-145 and VchSat07-36 exhibit a significantly lower AT content, i.e., 44.1%, and 16.7%, respectively (Table 2). Indeed, some heterochromatic (and rich in repetitive DNAs) microchromosome pairs are common in birds [65,66,67].

Comparative analyses between the sexes have demonstrated that satellite DNAs are of great importance for the differentiation of sex chromosomes [16,26,27,68,69,70,71,72,73]. The Z and W chromosomes of all birds are descended from the same ancestral autosomal pair, most likely between 60 and 100 million years ago [74]. Most bird species have a W chromosome that is frequently small and extremely heterochromatic [2,4,75,76,77,78,79,80]. However, an overview of bird’s sex chromosome evolution refuted the theory that W chromosomes gradually shrank during evolution, indicating their high plasticity [81,82]. Contrary to *J. jacana* [26], where the W chromosome was almost completely heterochromatic, the W chromosome in *V. chilensis* displayed an unusual lack of heterochromatin content. While our CGH tests revealed that the W chromosome is composed of many W-specific elements (Figure 4), only three sequences—VchSat01-179, VchSat02-5351, and (GAA)_n_—showed accumulation in the W chromosome (Figure 2). Therefore, other classes of repetitive DNA sequences, such as transposable elements (TEs), may represent the most prevalent type accumulated on the W chromosome, accounting for its molecular divergence to the Z chromosome. However, further research is required to validate this theory.

## 5. Conclusions

Characterizing the *V. chilensis* satellitome revealed the existence of seven satDNA families, with satellites predominantly having repeat units larger than 100 base pairs. Chromosomal mapping of these sequences showed that they are mainly present in centromeric regions, specifically on the W chromosome and microchromosomes. In conclusion, this study significantly contributed to a better understanding of repetitive sequences’ evolutionary and chromosomal organization in a neglected bird group.

## Figures and Tables

**Table 1:** List of species analyzed in this work with information about their sampling location, number, and sex of the individuals.

Species	Sampling Location	Number and Sex of Individuals
*J. jacana*	São Gabriel, Rio Grande do Sul, Brazil (30°18′07.2″ S 54°22′51.9″ W)	02♀ and 01♂
*V. chilensis*	São Gabriel, Rio Grande do Sul, Brazil (30°18′07.2″ S 54°22′51.9″ W)	01♀ and 01♂
*C. canutus*	Belém, Pará, Brazil (1°17′24.8″ S 48°26′29.9″ W)	01♀ and 01♂

**Table 2:** General features of *V. chilensis* (VCH) satellitome, including the repeat unit length (RUL), the abundance (%) of each satellite in the male and female genomes, and abundance in male/female proportion (%), divergence (%), and adenine plus thymine content (A + T%).

SatDNA	RUL (bp)	Abundance F (%)	Divergence F (%)	Abundance M (%)	Divergence M (%)	Abundance F/M (%)	A + T (%)
VchSat01-179	179	0.00566813534316	3.47	0.00734518650644	3.20	0.77	47.4%
VchSat02-5351	5351	0.00153609301945	3.92	0.000995645031422	3.39	1.54	54.0%
VchSat03-2447	2447	0.000786165893518	0.51	0.000954216629777	0.50	0.82	56.9%
VchSat04-145	145	0.000559347409739	11.61	0.000618731320616	11.50	0.9	44.1%
VchSat05-958	958	0.000419615999712	6.22	0.000496465807068	6.25	0.84	52.4%
VchSat06-234	234	0.000200179487108	6.99	0.000131052708244	10.13	1.52	37.6%
VchSat07-36	36	0.00000994875019722	6.29	0.00000619126971609	6.02	1.6	16.7%

## Data Availability

All the data supporting our findings are contained within the manuscript.

## Supplementary Materials

The following supporting information can be downloaded at: https://www.mdpi.com/article/10.3390/genes15020258/s1, Figure S1: Schematic representation of repetitive DNAs embedded in five VchSatDNAs (VchSat01-03; VchSat05 and VchSat06). Each class is depicted as a different color: Non-Long Terminal repeats (blue); Endogenous Retrovirus sequences (purple); Transposons (red); Long Terminal repeats (brown) and Interspersed repeats (black).
