# Supplementary figures and images for: Satellitome Analysis in the Southern Lapwing (Vanellus chilensis) Genome: Implications for SatDNA Evolution in Charadriiform Birds
Source: Genes (Basel). 2024 Feb 19;15(2):258. doi: 10.3390/genes15020258 (PMC10887557; doi:10.3390/genes15020258)

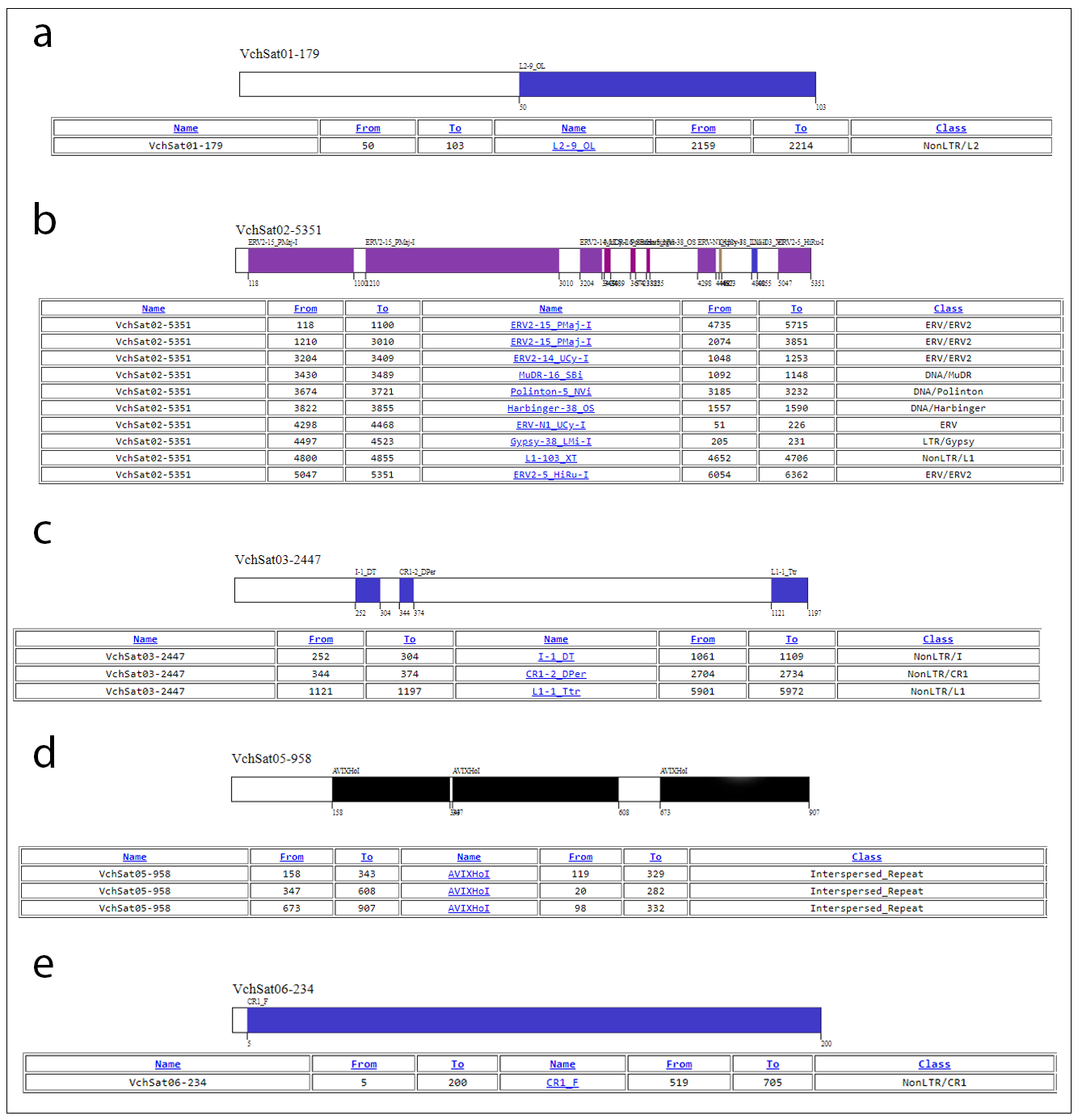

Supplement: Supplementary file 1 [file genes-15-00258-s001.zip › Supplementary figure 1.tif]
